# Knowledge, attitudes, and practice of pelvic floor dysfunction and pelvic floor ultrasound among women of childbearing age in Sichuan, China

**DOI:** 10.3389/fpubh.2023.1160733

**Published:** 2023-05-10

**Authors:** Xiaoli Wu, Xiaohong Yi, Xiu Zheng, Zeling Chen, Junxi Liu, Xiong Dai

**Affiliations:** Department of Ultrasound, Panzhihua Central Hospital, Panzhihua, Sichuan, China

**Keywords:** knowledge, attitudes, practice, pelvic floor disorders, pelvic floor ultrasound

## Abstract

**Objective:**

Pelvic floor dysfunction (PFD) is highly prevalent among women. Pelvic floor ultrasound (PFU) is a critical method for assessing PFD. This study examined the knowledge, attitudes, and practice (KAP) of women of childbearing age regarding PFD and PFU.

**Methods:**

This cross-sectional study was conducted between August 18, 2022, and September 20, 2022, in Sichuan, China. A total of 504 women of childbearing age participated in this study. A self-administered questionnaire was developed to assess KAP regarding PFD and PFU. Univariable and multivariable logistic regression analyses were conducted to assess the association between demographic characteristics and KAP.

**Results:**

The average scores for knowledge, attitudes, and practice were 12.53, 39.98, and 16.51 out of 17, 45, and 20, respectively. Despite adequate knowledge of PFD symptoms, aging-related risks, and PFD harms (correct rates > 80%), participants showed poor knowledge about the benefits of PFU, PFU types, and Kegel exercise (correct rates < 70%). High scores in knowledge and attitude (odds ratio = 1.23 and 1.11, *P* < 0.001 and *P* = 0.005, respectively) were independent predictors of good practice, while never having been pregnant (odds ratio = 0.10, *P* < 0.001), alcohol consumption (odds ratio = 0.09, *P* = 0.027), and not being diagnosed with PFD or an unclear diagnosis independently predicted poor practice (both odds ratio = 0.03, both *P* < 0.001).

**Conclusion:**

Women of childbearing age in Sichuan, China, showed moderate knowledge, positive attitude, and good practice regarding PFD and PFU. Knowledge, attitude, pregnancy history, alcohol consumption, and PFD diagnosis are associated with practice.

## Background

The term pelvic floor dysfunction (PFD) refers to a wide range of diseases resulting from the relaxation of the pelvic supports, including pelvic organ prolapse, stress urinary incontinence, overactive bladder, sexual dysfunction, rectal incontinence, and chronic pelvic pain (1). It is a health problem affecting women of all ages worldwide, with a prevalence between 25.0 and 60.2% (2, 3). There is a 20% lifetime risk of surgical intervention for pelvic organ prolapse or urinary incontinence (2, 4). Measures should be taken to reduce the risk of surgical intervention and improve quality of life (5).

Assessing the pelvic floor anatomy and function based on clinical examination alone is insufficient since it only describes surface anatomy rather than structural abnormalities. Currently, imaging plays an important role in investigating PFD (6, 7). Compared with other imaging techniques such as computed tomography and magnetic resonance imaging, pelvic floor ultrasound (PFU) offers the advantages of being a non-invasive, simple, inexpensive, and easily accessible assessment of pelvic floor muscle function (7, 8). As the main tool for the dynamic assessment of PFD, PFU allows the assessment of multiple anatomical and functional aspects simultaneously (9, 10). In addition to their prognostic value, PFU parameters can also predict the risk of PFD (11, 12), which can be used to guide pelvic floor muscle training (13).

It is essential for women of childbearing age to understand the harms of PFD and the importance of PFU in assessing PFD, but most studies that assessed the knowledge, attitudes, and practice (KAP) regarding PFD were conducted in western countries or among pregnant women or health workers (14–17), and no studies examined the KAP regarding PFU. Therefore, this study assessed the KAP regarding PFD and PFU among women of childbearing age in Sichuan, China, and the sociodemographic factors associated with KAP. The results may be shared through social media and medical practitioners to reduce the incidence of PFD and its harm to women of childbearing age.

## Methods

### Study design and subjects

This cross-sectional study was conducted at Panzhihua Central Hospital between August 18, 2022, and September 20, 2022. A total of 504 female outpatients and inpatients of childbearing age were recruited using convenience sampling. The inclusion criteria were (1) females between the ages of 18 and 50 and (2) willing to participate in this study. The patients who could not complete the questionnaire survey due to an inability to write or psychological diseases were excluded. This study was approved by the Research Ethics Committee of Panzhihua Central Hospital (approval No: pzhszxyyll-2022-18). All participants provided written informed consent before completing the questionnaire.

### Questionnaire

The questionnaire was designed by the investigators based on a previous study (14) and contained 47 questions in four categories. The final version was in Chinese. The pretest showed Cronbach's α of 0.90 and KMO of 0.94. The four categories included personal information (12 questions; Supplementary Table 1), knowledge (17 questions; Supplementary Table 2), attitudes (nine questions; Supplementary Table 3), and practice (nine questions; Supplementary Table 4). The knowledge category was scored from 0 to 17 points, with 1 point awarded for each correct answer and 0 for each wrong or unclear answer. The attitude category was scored from 9 to 45 points. For questions one to six and eight to nine, a five-point Likert scale was used (1 = strongly disagree, 2 = disagree, 3 = neutral, 4 = agree, 5 = strongly agree), with 5 points for a positive attitude and 1 point for a negative attitude. For question seven, 1 point awarded for “Both of them are unaccepted” and 5 point for other choices. The practice category was scored from 0 to 20. “Yes” to questions one to four received 1 point, whereas answers “no” received 0. “Never done” was given 1 point for question five, whereas other choices received 1 point each (1 point for each item selected, with a maximum score of 4 points). For questions 7 to 9, a five-point Likert scale was used (0 = strongly disagree, 1 = disagree, 2 = neither agree nor disagree/do not know, 3 = agree, 4 = strongly agree). A higher score indicates a higher level of KAP.

The paper version of the questionnaire was distributed when the patients visited the pelvic floor rehabilitation center and the ultrasound department of the hospital. QR codes were also provided to access the electronic version. The patients choosing to complete the paper version were provided with a private and quiet location to complete it. The electronic questionnaires were distributed via Wechat (Tencent, China) using a link created by the Wen-Juan-Xing online platform (Changsha Ranxing Information Technology Co., Ltd; https://www.wjx.cn/app/survey.aspx), an online questionnaire software platform.

### Statistical analysis

Without relevant literature on PFD and PUF KAP in the Chinese population, the study sample size was calculated with an anticipated proportion of PFD practice of 50%. At the 95% confidence level and 5% error margin, the required sample size was 384 (18).

The statistical analysis was performed using SPSS 26.0 (IBM, Armonk, NY, USA). The continuous data with a normal distribution were expressed as means ± standard deviations and analyzed using Student's *t*-test or ANOVA. The categorical data were expressed as frequency (percentage) and analyzed using the chi-square test. The Pearson correlation was used to analyze the correlation between the knowledge, attitude, and practice scores. Participants with the highest tertile knowledge, attitude, and practice scores were considered to have a good KAP. Univariable logistic regression analysis of knowledge, attitude, and practice was performed first. We used structural equation modeling (SEM) to evaluate the path association between baseline characteristics and KAP. Except for knowledge included in attitude and knowledge and attitude included in practice, the coefficient ≥1 between baseline characteristics and KAP in SEM path analysis was included in multivariable logistic regression analysis (Supplementary Figure 1). The statistical tests were all two-sided, and a *P*-value of < 0.05 was considered statistically significant.

## Results

Table 1 shows the sociodemographic characteristics and KAP scores of the participants. The participants were mostly 18–27 years old (35.9%), married (71.0%), sexually active (83.1%), with no pregnancies (30.3%), with one child (38.7%), vaginal delivery (32.9%), with bachelor's degree or associate degree (62.3%), with professional jobs (36.7%), urban residency (65.5%), an income of 2,000–5,000 (43.5%), not smoking (91.7%), not drinking (89.9%), and without PFD (59.5%).

**Table 1:** Participants' demographics and knowledge, attitude, and practice scores regarding pelvic floor dysfunction and pelvic floor ultrasound.

**Characteristics**		**Knowledge**			**Attitude**			**Practice**		
	***N*** = **504**	**Mean**	**SD**	***P***	**Mean**	**SD**	***P***	**Mean**	**SD**	***P***
Total		12.53	5.42		39.98	4.84		16.51	6.60	
**Age (years)**				< 0.001			< 0.001			< 0.001
18–27	181 (35.9)	12.73	5.49		39.22	5.12		14.12	5.32	
28–33	151 (30.0)	13.70	4.57		41.24	4.07		17.60	6.92	
34–50	172 (34.1)	11.30	5.79		39.69	4.95		18.06	6.86	
**Marital status**				0.141			0.140			< 0.001
Single	130 (25.8)	13.18	5.35		39.29	5.20		12.83	4.18	
Married	358 (71.0)	12.37	5.37		40.19	4.66		17.89	6.89	
Divorced/Widowed	16 (3.2)	10.75	6.69		40.94	5.29		15.56	4.24	
**Sexually active**				0.069			0.285			< 0.001
Yes	419 (83.1)	12.33	5.47		40.09	4.67		39.98	4.84	
No	85 (16.9)	13.51	5.11		39.47	5.60		12.76	4.36	
**Gravidity**				0.398			0.207			< 0.001
0	153 (30.3)	12.85	5.64		39.30	5.14		12.64	3.89	
1	141 (28.0)	12.91	5.13		40.21	4.61		17.36	6.84	
2	89 (17.7)	11.92	5.37		40.18	4.69		17.96	7.01	
≥3	121 (24.0)	12.14	5.51		40.44	4.76		19.35	6.58	
**Parity**				0.300			0.388			< 0.001
0	193 (38.3)	12.84	5.54		39.54	5.15		12.83	3.98	
1	195 (38.7)	12.59	5.35		40.14	4.81		18.53	6.91	
2	107 (21.2)	12.09	5.32		40.50	4.28		19.26	6.91	
≥3	9 (1.8)	9.78	5.43		40.00	4.61		19.00	6.12	
**Mode of delivery**				0.148			0.209			< 0.001
Vaginal delivery	166 (32.9)	12.15	5.32		40.24	4.39		19.26	6.94	
Cesarean delivery	129 (25.6)	12.84	5.40		40.50	5.04		18.53	6.93	
Vaginal and cesarean deliveries	17 (3.4)	10.12	5.80		39.00	3.84		15.76	5.25	
Never pregnant	192 (38.1)	12.87	5.45		39.50	5.11		12.84	3.98	
**Education**				< 0.001			< 0.001			0.083
Primary or middle school	85 (16.9)	9.78	5.70		38.66	4.79		17.33	6.68	
High school or secondary school	82 (16.3)	9.68	6.30		38.35	5.29		15.72	6.43	
Bachelor's or associate degree	314 (62.3)	13.84	4.54		40.74	4.48		16.69	6.66	
Master's or doctoral degree	23 (4.5)	14.96	4.25		40.35	5.79		13.78	5.60	
**Occupation**				< 0.001			0.014			0.709
Leaders of party–mass organization of state organs, enterprises, and institutions	14 (2.8)	13.14	4.72		40.07	5.69		19.50	7.16	
Professional jobs, such as a teacher, doctor, engineer, and writer	185 (36.7)	14.56	4.18		40.88	4.66		16.27	6.42	
Non-professional jobs	56 (11.1)	11.59	5.79		40.75	4.17		16.07	6.79	
Commercial and service industry jobs	35 (6.9)	10.43	6.50		39.14	5.98		17.20	6.24	
Jobs related to agriculture, forestry, animal husbandry, fisheries, and water resources production	13 (2.6)	11.23	4.94		40.15	3.29		16.23	6.55	
Jobs related to equipment operation or transportation	7 (1.4)	9.29	6.42		39.29	4.19		16.43	8.06	
Others	194 (38.5)	11.41	5.64		39.07	4.86		16.55	6.73	
**Residency**				0.001			0.003			0.042
Rural	146 (29.0)	11.21	5.79		39.15	4.81		15.57	6.07	
Urban	330 (65.5)	13.15	5.11		40.49	4.86		17.04	6.81	
Suburb	28 (5.5)	12.11	5.90		38.32	3.72		15.14	6.29	
**Household's monthly capita income**				0.005			0.105			0.155
< 2,000	64 (12.7)	12.47	5.26		39.70	4.46		18.06	7.19	
2,000–5,000	219 (43.5)	11.68	5.79		39.42	4.94		15.87	6.59	
5,000–10,000	156 (30.9)	13.37	5.08		40.46	5.01		16.47	6.25	
10,000–20,000	49 (9.7)	14.18	3.87		41.06	4.21		17.47	6.43	
>20,000	16 (3.2)	11.13	6.17		40.88	4.23		16.56	7.63	
**Smoking**				0.006			0.476			0.720
No	462 (91.7)	12.76	5.24		40.00	4.70		16.57	6.67	
Quit smoking	29 (5.7)	10.17	6.78		39.17	7.07		16.07	6.31	
Yes	13 (2.6)	9.62	6.54		41.08	3.66		15.23	5.02	
**Alcohol drinking**				0.004			0.628			0.166
No	453 (89.9)	12.76	5.32		40.00	4.83		16.67	6.53	
Quit drinking	34 (6.7)	11.41	5.85		40.29	5.17		15.79	7.60	
Yes	17 (3.4)	8.65	5.87		38.94	4.47		13.76	6.08	
**Pelvic floor dysfunction diagnosed**				< 0.001			< 0.001			< 0.001
Yes, but not yet treated	77 (15.3)	15.00	3.10		41.97	3.61		24.44	3.87	
Yes, and has been treated	35 (6.9)	11.91	5.12		40.49	4.18		21.91	5.92	
No	300 (59.5)	12.69	5.38		39.75	4.85		14.69	5.59	
Unclear	92 (18.3)	10.17	6.20		38.89	5.45		13.75	5.45	

SD, standard deviation.

Participants with the highest tertile knowledge, attitude, and practice scores were considered to have moderate knowledge, positive attitude, and good practice. The corresponding cut-off scores were 16, 43, and 18. The knowledge, attitude, and practice scores were 12.53 ± 5.42 (on a maximum of 17: 73.7%), 39.98 ± 4.84 (on a maximum of 45: 88.9%), and 16.51 ± 6.60 (on a maximum of 20: 82.6%), respectively, suggesting that participants have moderate knowledge, positive attitudes, and practiced well regarding PFD and PFU. The proportion of participants with moderate knowledge, positive attitude, and good practice is 36.5 (184/504), 36.1 (182/504), and 33.3 (168/504), respectively.

The knowledge scores were correlated with the attitude (*r* = 0.507, *P* < 0.001) and practice (*r* = 0.431, *P* < 0.001) scores. The attitude scores were correlated with the practice scores (*r* = 0.408, *P* < 0.001) (Table 2).

**Table 2:** Correlation analysis.

	**Knowledge**	**Attitude**	**Behavior**
Knowledge	1	/	/
Attitude	0.507 (*P* < 0.001)	1	/
Practice	0.431 (*P* < 0.001)	0.408 *(P* < 0.001)	1

**Figure 1:**
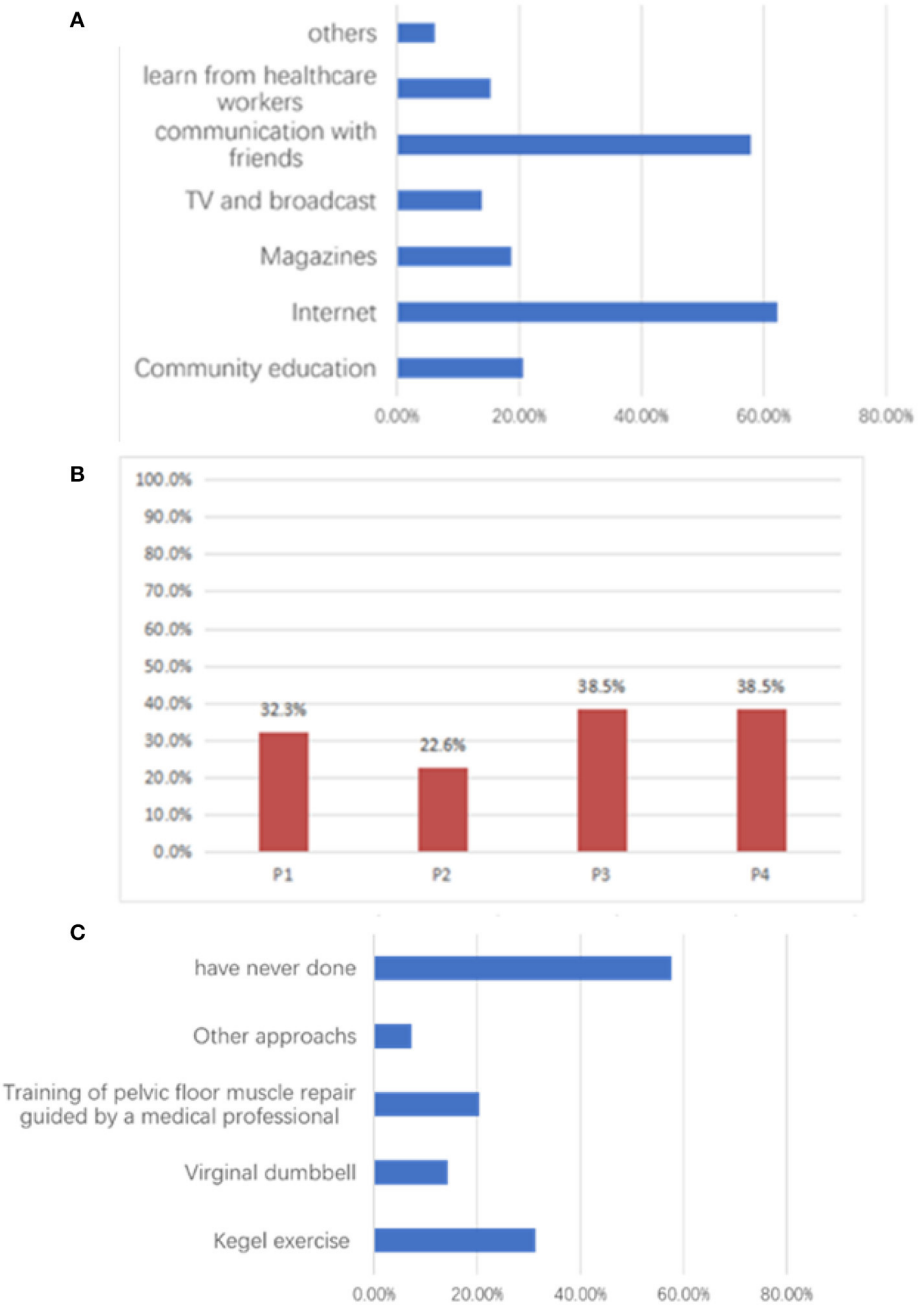
The participants' practices regarding pelvic floor dysfunction and pelvic floor ultrasound. **(A)** Percentage of yes responses to practice questions. P1: Have been assessed for pelvic floor muscle function; P2: Have had a pelvic floor ultrasound; P3: Have proactively learned about pelvic floor dysfunction; P4: Have proactively sought medical attention when necessary. **(B)** Information sources about women's healthcare. **(C)** Exercises performed by participants to strengthen the pelvic floor.

Higher knowledge scores were observed in younger age groups (*P* < 0.001), with higher education (*P* < 0.001), with professional jobs (*P* < 0.001), urban residency (*P* = 0.001), higher income (*P* = 0.005), not smoking (*P* = 0.006), not drinking (*P* = 0.004), and with diagnosed and treated PFD (*P* < 0.001). There was good knowledge among participants regarding PFD symptoms, the increased risk of PFD associated with aging, and the harms caused by PFD (all correct rates > 80%), but knowledge regarding the benefits of PFU (correct rate = 60.9%), PFU types (correct rate = 61.1%), and Kegel motion (correct rate = 65.1%) were lacking (Table 3). Knowledge was primarily acquired through the internet, followed by communication with friends and community education (Figure 1A).

**Table 3:** The correct rate of knowledge questionnaire.

**Knowledge**	**Correct rate**
Types of pelvic floor dysfunction	72.4
Symptoms of pelvic floor dysfunction	82.9
The risk of pelvic floor dysfunction increases with age.	81.2
Pregnancy and childbirth may lead to pelvic floor dysfunction.	78.0
The risk factors for pelvic floor dysfunction include sedentary lifestyles, obesity, long–term restraint of the waist, constipation, and diabetes.	71.2
Pregnancy induces pelvic floor damage	80.0
After delivery, when should pelvic floor muscle function tests be performed?	80.4
Pelvic floor exercises before pregnancy may help prevent postpartum urine leakage.	70.6
Pelvic floor dysfunction can cause physical and mental harm to women.	85.3
Pelvic floor exercises can reduce urine leakage.	78.6
Kegel exercises	65.1
Rehabilitation of pelvic floor dysfunction	69.8
Benefits of pelvic floor ultrasound	60.9
Common types of pelvic floor ultrasound	61.1
The diagnostic role of regular pelvic floor ultrasound in pelvic floor dysfunction	76.0
A pelvic floor ultrasound is essential before pelvic floor surgery.	69.4
Application of pelvic floor ultrasound	70.2

Higher attitude scores were observed in participants of 28–33 years (*P* < 0.001), with higher education (*P* < 0.001), with non-commercial/non-service jobs (*P* = 0.014), urban residency (*P* = 0.003), and with PFD diagnosis and treatment (*P* < 0.001). In the attitude assessment, over 90% of the participants wanted to learn more about PFD (91.7%) and specific methods of pelvic floor exercises (92.3%), believed PFD could affect their life (92.1%), work (90.1%), and couples' relationship (89.9%), and were willing to undergo PFU (93.8%). Most participants wanted to know their pelvic floor function status (83.5%) and believed that women of all ages should train their pelvic floor muscles (85.5%).

Higher practice scores were observed in older age groups (*P* < 0.001), married women (*P* < 0.001), sexually active women (*P* < 0.001), with higher numbers of children (*P* < 0.001), with vaginal delivery (*P* < 0.001), urban residency (*P* = 0.042), and with PFD diagnosis and treatment (*P* < 0.001). While the practice assessment score was 16.51 out of 20, < 40% of participants had been assessed for pelvic floor muscle function, undergone PFU, or proactively sought medical advice when concerned about PFD (Figure 1B). Most participants had never exercised their pelvic floor muscles (Figure 1C).

We used SEM to screen variables that were significant in the initial univariable logistic regression analysis (Table 4). We included baseline characteristics with coefficients ≥1 in the multivariable logistic regression analysis except for knowledge that was already included in attitude analysis and knowledge and attitude that were already included in practice analysis (Supplementary Figure 1). The results of the multivariable logistic regression analysis showed that education level and PFD diagnosis were significantly associated with knowledge about PFD. Compared to those with primary or middle school education, individuals with a bachelor's or associate degree (odds ratio = 4.75, 95% confidence interval: 2.52, 8.98, *P* < 0.001) and those with a Master's or Doctoral degree (odds ratio = 6.54, 95% confidence interval: 2.34, 18.25, *P* < 0.001) had significantly higher odds of having knowledge about PFD. However, those who had not been diagnosed with PFD (odds ratio = 0.47, 95% confidence interval: 0.27, 0.82, *P* = 0.008) or had an unclear diagnosis (odds ratio = 0.31, 95% confidence interval: 0.154, 0.62, *P* = 0.001) had significantly lower odds of having knowledge about it than those who had been diagnosed but not yet treated (Table 5). In addition, knowledge was significantly positively associated with attitude (odds ratio = 1.15, 95% confidence interval: 1.10, 1.21, *P* < 0.001). Marital status was also significantly associated with attitude, with married individuals having an odds ratio of 1.82 (95% confidence interval: 1.10, 3.00; *P* = 0.019) and divorced/widowed individuals having an odds ratio of 4.42 (95% confidence interval: 1.31, 14.97; *P* = 0.017) compared to single individuals. Regarding education, individuals with a bachelor's or associate degree have a significant positive association with attitude (odds ratio = 1.93, 95% confidence interval: 1.04, 3.58; *P* = 0.038). However, no significant associations were found for individuals with a high school or secondary school education or a master's or doctoral degree (Table 6). Furthermore, knowledge (odds ratio = 1.23, 95% confidence interval: 1.13, 1.33, *P* < 0.001) and attitude (odds ratio = 1.11, 95% confidence interval: 1.03, 1.20; *P* = 0.005) were significantly positively associated with practice. On the other hand, never pregnant individuals (odds ratio = 0.10, 95% confidence interval: 0.04, 0.24; *P* < 0.001), alcohol drinkers (odds ratio = 0.09, 95% confidence interval: 0.01, 0.76; *P* = 0.027), and those not diagnosed with PFD (odds ratio = 0.03, 95% confidence interval: 0.01, 0.10; *P* < 0.001) or with an unclear diagnosis (odds ratio = 0.03, 95% confidence interval: 0.01, 0.11; *P* < 0.001) had a significant negative association with practice (Table 7).

**Table 4:** Univariable logistic regression analysis with knowledge, attitude and practice as the dependent variable.

**Factors**	**Knowledge**		**Attitude**		**Practice**	
	**OR (95%CI)**	***P***	**OR (95%CI)**	***P***	**OR (95%CI)**	***P***
Knowledge score	–	–	1.168 (1.115–1.224)	< 0.001	1.195 (1.134–1.259)	< 0.001
Attitude score	–	–	–	–	1.184 (1.127–1.244)	< 0.001
**Age (Grouping in tertiles)**						
18–27	REF	–	REF	–	REF	–
28–33	1.231 (0.794–1.910)	0.353	2.144 (1.362–3.375)	0.001	3.361 (2.010–5.622)	< 0.001
34–50	0.614 (0.392–0.961)	0.033	1.329 (0.848–2.083)	0.215	4.558 (2.711–7.496)	< 0.001
**Marital status**						
Single	REF	–	REF	–	REF	–
Married	0.634 (0.421–0.954)	0.029	1.558 (1.007–2.411)	0.046	10.265 (5.053–20.853)	< 0.001
Divorced/Widowed	0.564 (0.186–1.716)	0.313	2.514 (0.878–7.193)	0.086	4.481 (1.199–16.755)	0.026
**Sexually active**						
Yes	REF		REF		REF	
No	1.698 (1.060–2.719)	0.028	0.900 (0.551–1.470)	0.675	8.30 (3.537–19.476)	< 0.001
**Gravidity**						
0	REF	–	REF	–	REF	–
1	0.705 (0.441–1.129)	0.145	1.532 (0.946–2.484)	0.083	9.636 (4.530–20.497)	< 0.001
2	0.530 (0.303–0.925)	0.025	1.371 (0.789–2.380)	0.263	12.480 (5.647–27.581)	< 0.001
≥3	0.658 (0.402–1.078)	0.096	1.477 (0.893–2.443)	0.128	19.852 (9.257–42.571)	< 0.001
**Parity**						
0	REF	–	REF	–	REF	–
1	0.809 (0.537–1.217)	0.309	1.409 (0.929–2.136)	0.107	13.472 (7.045–25.765)	< 0.001
2	0.603 (0.364–0.997)	0.049	1.164 (0.707–1.915)	0.551	17.854 (8.891–35.850)	< 0.001
≥3	0.177 (0.022–1.440)	0.105	1.690 (0.439–6.514)	0.446	30.167 (6.704–135.742)	< 0.001
**Mode of delivery**						
Vaginal delivery	REF	–	REF	–	REF	–
Cesarean delivery	1.349 (0.834–2.184)	0.223	1.443 (0.902–2.308)	0.126	0.826 (0.521–1.309)	0.416
Vaginal and cesarean deliveries	1.33 (0.017–1.031)	0.054	0.379 (0.105–1.370)	0.139	0.266 (0.083–0.850)	0.026
Never pregnant	1.523 (0.986–2.352)	0.058	0.823 (0.530–1.276)	0.383	0.058 (0.030–0.112)	< 0.001
**Education**						
Primary or middle school	REF	–	REF	–	REF	–
High school or secondary school	1.131 (0.518–2.469)	0.757	0.857 (0.418–1.758)	0.674	0.632 (0.335–1.191)	0.156
Bachelor's or associate degree	3.803 (2.087–6.933)	< 0.001	2.329 (1.355–4.000)	0.002	0.664 (0.407–1.085)	0.102
Master's or doctoral degree	5.091 (1.891–13.702)	0.010	1.333 (0.483–3.682)	0.579	0.204 (0.056–0.740)	0.016
**Occupation**						
Leaders of party–mass organization of state organs, enterprises, and institutions	REF	–	REF	–	REF	–
Professional jobs, such as a teacher, doctor, engineer, and writer	1.984 (0.641–6.146)	0.235	0.762 (0.257–2.260)	0.624	0.494 (0.163–1.494)	0.212
Non-professional jobs	0.853 (0.250–2.913)	0.799	0.647 (0.199–2.099)	0.468	0.581 (0.175–1.934)	0.376
Commercial and service industry jobs	0.825 (0.224–3.044)	0.773	0.591 (0.169–2.067)	0.410	0.889 (0.253–3.121)	0.854
Jobs related to agriculture, forestry, animal husbandry, fisheries, and water resources production	0.540 (0.100–2.930)	0.475	0.300 (0.057–1.581)	0.156	0.833 (0.179–3.884)	0.816
Jobs related to equipment operation or transportation	0.000	0.999	0.167 (0.016–1.769)	0.137	1.778 (0.284–11.120)	0.538
Others	0.625 (0.200–1.953)	0.419	0.416 (0.136–1.210)	0.106	0.787 (0.262–2.359)	0.669
**Residency**						
Rural	REF	–	REF	–	REF	–
Urban	1.671 (1.098–2.545)	0.017	2.171 (1.409–3.344)	0.000	1.463 (0.957–2.238)	0.079
Suburb	1.173 (0.491–2.801)	0.719	0.640 (0.227–1.806)	0.399	0.854 (0.337–2.160)	0.738
**Household's monthly capita income**						
< 2,000	REF	–	REF	–	REF	–
2,000–5,000	0.949 (0.520–1.733)	0.865	0.970 (0.531–1.770)	0.920	0.440 (0.249–0.777)	0.005
5,000–10,000	1.935 (1.046–3.579)	0.035	1.700 (0.918–3.148)	0.091	0.487 (0.269–0.884)	0.018
10,000–20,000	1.517 (0.689–3.300)	0.293	1.517 (0.698–3.300)	0.293	0.674 (0.317–1.435)	0.306
>20,000	1.000 (0.307–3.261)	1.000	1.711 (0.558–5.246)	0.347	0.484 (0.151–1.552)	0.222
**Smoking**						
No	REF	–	REF	–	REF	–
Quit smoking	0.879 (0.400–1.934)	0.749	1.696 (0.799–3.601)	0.169	0.883 (0.393–1.984)	0.763
Yes	0.139 (0.018–1.080)	0.059	0.808 (0.245–2.663)	0.726	0.588 (0.160–2.169)	0.426
Alcohol drinking						0.198
No	REF	–	REF	–	REF	–
Quit drinking	0.674 (0.315–1.444)	0.311	1.222 (0.601–2.483)	0.580	0.919 (0.437–1.935)	0.825
Yes	0.101 (0.013–0.770)	0.027	0.374 (0.106–1.321)	0.127	0.256 (0.058–1.135)	0.073
Pelvic floor dysfunction diagnosed		0.016				
Yes, but not yet treated	REF	–	REF	–	REF	–
Yes, and has been treated	0.522 (0.225–1.212)	0.130	0.508 (0.22–1.164)	0.110	0.211 (0.070–0.641)	0.006
No	0.708 (0.427–1.173)	0.180	0.502 (0.302–0.833)	0.008	0.019 (0.008–0.047)	< 0.001
Unclear	0.358 (0.186–0.690)	0.002	0.449 (0.240–0.840)	0.012	0.018 (0.007–0.048)	< 0.001

OR, odds ratio.

**Table 5:** Multivariable logistic regression with knowledge as the dependent variable.

	**OR (95% CI)**	***P***
**Education**		
Primary or middle school	REF	
High school or secondary school	1.42 (0.64, 3.17)	0.390
Bachelor's or associate degree	4.75 (2.52, 8.98)	< 0.001
Master's or doctoral degree	6.54 (2.34, 18.25)	< 0.001
**Smoking**		
No	REF	
Quit smoking	1.34 (0.54, 3.35)	0.527
Yes	0.25 (0.03, 2.23)	0.212
**Alcohol drinking**		
No	REF	
Quit drinking	0.67 (0.28, 1.59)	0.365
Yes	0.13 (0.02, 1.11)	0.062
**Pelvic floor dysfunction diagnosed**		
Yes, but not yet treated	REF	
Yes, and has been treated	0.69 (0.27, 1.73)	0.427
No	0.47 (0.27, 0.82)	0.008
Unclear	0.31 (0.154, 0.62)	0.001

**Table 6:** Multivariable logistic regression with attitude as the dependent variable.

	**OR (95% CI)**	***P***
Knowledge	1.15 (1.10, 1.21)	< 0.001
**Marital status**		
Single	REF	
Married	1.82 (1.10, 3.00)	0.019
Divorced/Widowed	4.42 (1.31, 14.97)	0.017
**Education**		
Primary or middle school	REF	
High school or secondary school	0.91 (0.42, 1.96)	0.809
Bachelor's or associate degree	1.93 (1.04, 3.58)	0.038
Master's or doctoral degree	1.28 (0.41, 3.96)	0.670
**Pelvic floor dysfunction diagnosed**		
Yes, but not yet treated	REF	
Yes, and has been treated	0.75 (0.31, 1.82)	0.520
No	0.66 (0.37, 1.18)	0.165
Unclear	0.82 (0.40, 1.66)	0.579

**Table 7:** Multivariable logistic regression with practice as the dependent variable.

	**OR (95% CI)**	***P***
Knowledge	1.23 (1.13, 1.33)	< 0.001
Attitude	1.11 (1.03, 1.20)	0.005
**Sexually active**		
Yes	REF	
No	0.86 (0.28, 2.65)	0.797
**Mode of delivery**		
Vaginal delivery	REF	
Cesarean delivery	1.02 (0.52, 2.01)	0.957
Vaginal and cesarean deliveries	0.41 (0.09, 1.81)	0.240
Never pregnant	0.10 (0.04, 0.24)	< 0.001
**Residency**		
Rural	REF	
Urban	1.61 (0.82, 3.19)	0.169
Suburb	1.71 (0.44, 6.62)	0.436
**Alcohol drinking**		
No	REF	
Quit drinking	0.58 (0.14, 2.51)	0.469
Yes	0.09 (0.01, 0.76)	0.027
**Pelvic floor dysfunction diagnosed**		
Yes, but not yet treated	REF	
Yes, and has been treated	0.48 (0.12, 1.85)	0.284
No	0.03 (0.01, 0.10)	< 0.001
Unclear	0.03 (0.01, 0.11)	< 0.001

## Discussion

It is the first study to examine the KAP regarding PFD and PFU among Chinese women of childbearing age. Twenty-two percent of the respondents have been diagnosed with PFD, comparable to the previously reported prevalence of PFD in China (19, 20). The KAP scores indicated moderate knowledge, positive attitude, and good practice among participants. Significant and positive correlations were found between knowledge and attitude, knowledge and practice, and attitude and practice. The knowledge and attitude scores were independent positive predictors of good practice, whereas the absence of a PFD diagnosis was an independent negative predictor.

According to our study, the knowledge scores were significantly higher among patients between 28 and 33 years old, with higher education, professional jobs, living in urban areas, having a relatively high income, being non-drinkers, non-smokers, and having pelvic floor dysfunction diagnosed but not yet treated. People who do not drink or have quit drinking alcohol may be more knowledgeable about PFD due to their overall increased health consciousness and interest in maintaining a healthy lifestyle. This heightened awareness may prompt them to learn about various health conditions, including PFD, and understand the impacts of certain lifestyle choices on their health. Additionally, those who have experienced PFD symptoms may be more likely to abstain from alcohol, as they recognize its potential to exacerbate the condition. Over 60% of participants used the Internet as their primary source of healthcare information, possibly because women with these features have easier access to the Internet. Based on a study of pregnant women in Singapore, the mean knowledge scores for PFD significantly increased with educational level, reaching the highest among women aged 36 to 41 (16). Women with higher socioeconomic status are more likely to have a higher health literacy (21), supporting the present study. It is reasonable to expect that women would gain knowledge about PFD during previous pregnancies. However, both studies, despite the non-significant results in ours, showed that nulliparous women knew more about PFD and PFU than multiparous women. Therefore, higher parity may not necessarily result in greater knowledge because childcare commitments may reduce women's attention to their health. In addition, early childbearing may be associated with lower levels of education and employment for women. All pregnant women should receive antenatal education regardless of parity.

A pelvic floor muscle training program such as Kegel exercises strengthens pelvic floor muscles and prevents urinary and fecal incontinence (22–25). Daly et al. (26) found that 41% of pre-pregnant women in Dublin, Ireland learned pelvic floor muscle exercises, yet only 30% of them performed the exercises. An United States-based study involving 3,733 females aged 18 years or older found that 92.5% knew about Kegel exercises, whereas only 42.6% performed them (27). Similarly, In our study, while over 60% of participants had correct knowledge about pelvic floor muscle exercises, most had never practiced them. As reported, women with pelvic organ prolapse were more likely to exercise their pelvic floor muscles than those without pelvic organ prolapse (34.8 vs. 16.4%), (27) which is consistent with our finding that not being diagnosed with PFD independently predicted low practice scores. These findings indicate that women with symptomatic PFD are more likely to exercise pelvic floor muscles. In the present study, women with pregnancies and children were likelier to have a high practice score regarding PFD. Having been exposed to the risk of PFD or having developed PFD can enhance the women's practice to avoid PFD or avoid PFD progression. Considering the impact of PFD on sexuality (28), it is not surprising that sexually active women have better practice scores. Since vaginal delivery is a major risk factor for PFD (29), it is also not surprising that women with vaginal delivery have better practice scores regarding PFD and PFU.

Knowledge, attitude, and practice were positively correlated, suggesting that women with higher knowledge and attitude are more likely to practice. In our study, participants acquired knowledge primarily through the internet, followed by communication with friends. Only 20% of respondents gained knowledge from community education and even fewer from healthcare workers, suggesting a lack of education from both. It has been reported that exercise education classes significantly improved the KAP of pelvic floor muscle exercise in antenatal and postpartum women (30). An education program about pelvic floor muscles improved women's knowledge about the pelvic floor (15). In a KAP study about pelvic floor muscle training in people with spinal cord injury, most respondents agreed or strongly agreed that they would be comfortable talking about pelvic floor muscle training with a healthcare professional rather than a friend. Only 28% of respondents, however, indicated that a healthcare professional had discussed pelvic floor muscle training with them since the injury (31). Therefore, engaging the community and health workers in educating people about PFD and PFU is important. Not surprisingly, women with a diagnosis and who were treated for PFD had better KAP than those without diagnosis/treatment. Patients gain information from the healthcare providers when they are diagnosed, and they are prone to seek information and adopt favorable attitudes and practices.

The SEM analysis identified significant variables in the initial univariable logistic regression analysis and found that education level and PFD diagnosis were significantly associated with knowledge about PFD. Knowledge was positively associated with attitude and practice, while never being pregnant, alcohol drinking, and not being diagnosed with PFD or having an unclear diagnosis were negatively associated with practice. Married individuals and those with a bachelor's or associate degree had a positive association with attitude. These findings suggest that interventions targeting education and PFD diagnosis may improve knowledge and practice regarding PFD, while considering factors such as marital status and education level may improve attitudes toward PFD.

Since this was a cross-sectional study, causality cannot be inferred because a temporal sequence cannot be established. In this study, data were collected by self-reporting, which might be less reliable than medical records and laboratory measurements due to self-reporting bias. In addition, as this study was conducted in Sichuan, China, the results do not reflect the KAP of PFD and PFU globally. More studies in more areas with larger sample sizes are needed to understand better the KAP of PFD and PFU around the world.

## Conclusion

In this study, the KAP of PFD and PFU was assessed for the first time in women of childbearing age in China. While participants showed a positive attitude and good practice toward PFD and PFU, further efforts are needed to improve their knowledge.

## Data availability statement

The original contributions presented in the study are included in the article/Supplementary material, further inquiries can be directed to the corresponding author.

## Ethics statement

The studies involving human participants were reviewed and approved by Panzhihua Central Hospital Research Ethics Committee (Ethical approval No: pzhszxyyll-2022-18). Written informed consent to participate in this study was provided by the participants' legal guardian/next of kin.

## Author contributions

XW carried out the studies, participated in collecting data, and drafted the manuscript. XY, XZ, and XW performed the statistical analysis and participated in its design. ZC, JL, and XD helped to draft the manuscript. All authors read and approved the final manuscript.
