# Supplementary material for: Knowledge, attitudes, and practice of pelvic floor dysfunction and pelvic floor ultrasound among women of childbearing age in Sichuan, China
Source: Front Public Health. 2023 May 10;11:1160733. doi: 10.3389/fpubh.2023.1160733 (PMC10206020; doi:10.3389/fpubh.2023.1160733)
Supplement: Supplementary file 1 [file Image_1.pdf]

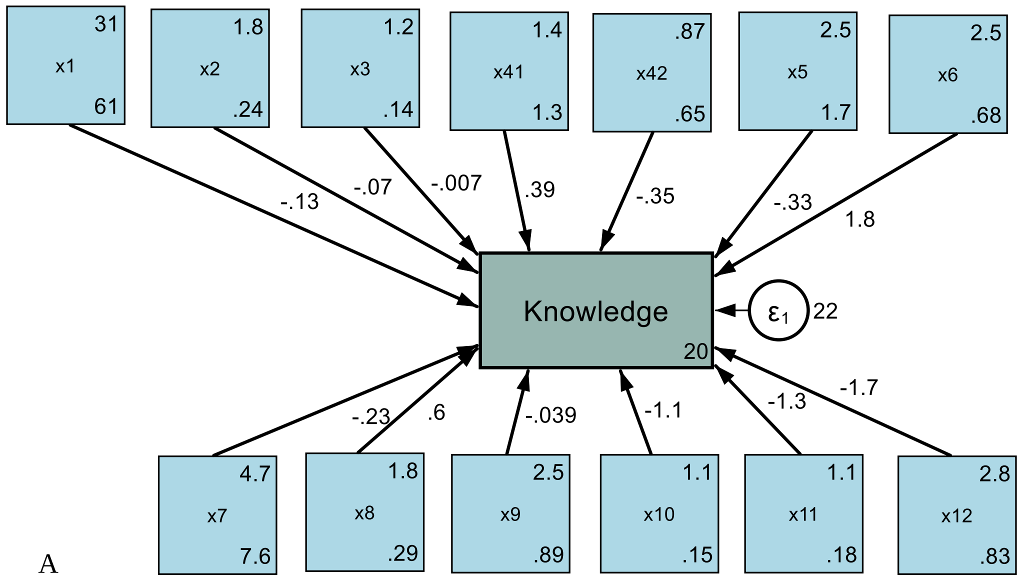

x1
31
61
x2
1.8
.24
x3
1.2
.14
x41
1.4
1.3
x42
.87
.65
x5
2.5
1.7
x6
2.5
.68
-.13
-.07
-.007
.39
-.35
-.33
1.8
Knowledge
20
ε1
22
-.23
.6
-.039
-1.1
-1.3
-1.7
x7
4.7
7.6
x8
1.8
.29
x9
2.5
.89
x10
1.1
.15
x11
1.1
.18
x12
2.8
.83

A

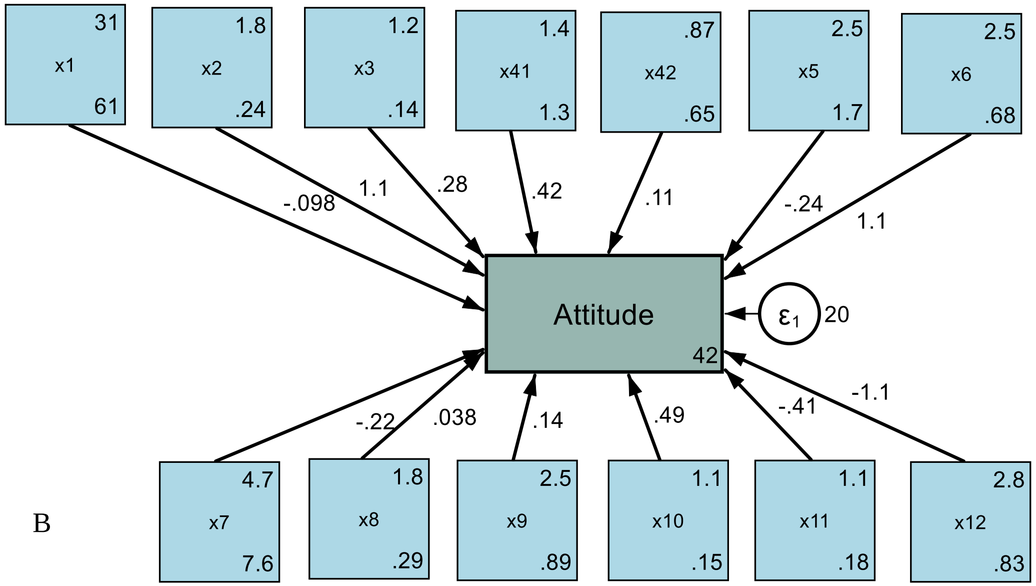
31
x1
61
1.8
x2
.24
1.2
x3
.14
1.4
x41
1.3
.87
x42
.65
2.5
x5
1.7
2.5
x6
.68
-.098
1.1
.28
.42
.11
-.24
1.1
Attitude
42
ε1
20
-.22
.038
.14
.49
-.41
-1.1
4.7
x7
7.6
1.8
x8
.29
2.5
x9
.89
1.1
x10
.15
1.1
x11
.18
2.8
x12
.83

B

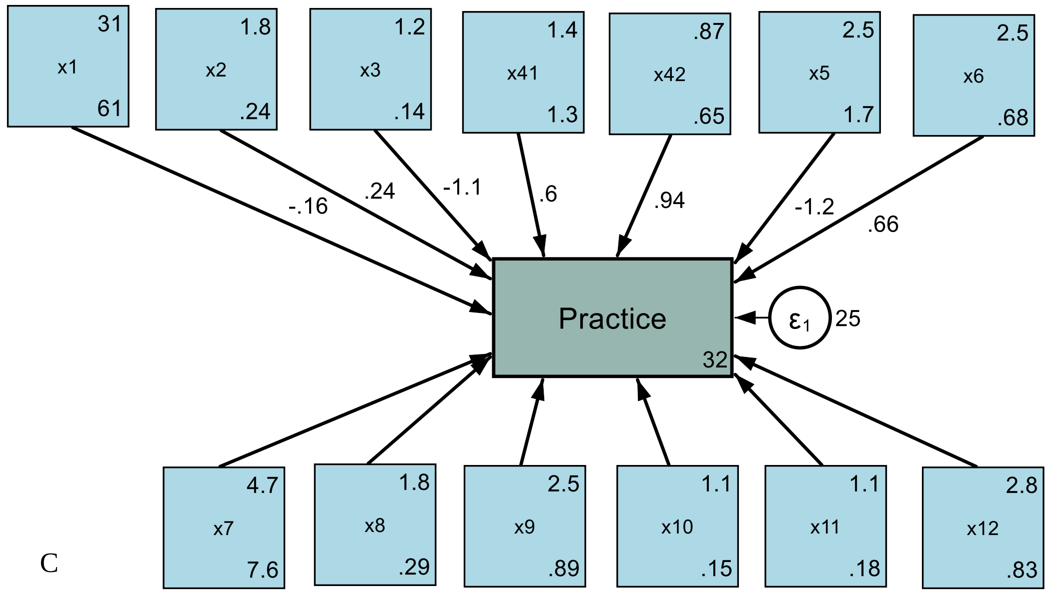

C

Figure S1. The SEM model of knowledge (A), attitude (B) and practice (C). x1: Age; x2: Marital status; x3: Sexually active; x41: Gravidity; x42: Parity; x5: Mode of delivery; x6: Education; x7: Occupation; x8: Residency; x9: Household's monthly capita income; x10: Smoking; x11: Alcohol drinking; x12: Pelvic floor dysfunction diagnosed.
