# Supplementary material for: Knowledge, attitudes, and practice of pelvic floor dysfunction and pelvic floor ultrasound among women of childbearing age in Sichuan, China
Source: Front Public Health. 2023 May 10;11:1160733. doi: 10.3389/fpubh.2023.1160733 (PMC10206020; doi:10.3389/fpubh.2023.1160733)
Supplement: Supplementary file 2 [file Table_1.DOCX]

| **Tabel S1 Personal information** | |
| --- | --- |
| **1. Age：** **years old** | |
| **2. Marital status** | a. Single  b. Married  c. Divorced  d. Widowed |
| **3. Sexually active** | a. Yes  b. No |
| **4.** **Gravidity** **, Parity** | |
| **5. Mode of delivery** | a. Vaginal delivery  b. Cesarean delivery  c. Vaginal and cesarean deliveries  d. Never pregnant |
| **6. Education** | a. Primary or middle school  b. High school or secondary school  c. Bachelor's or associate degree  d. Master's or doctoral degree |
| **7. Occupation** | a. Leaders of party-mass organization of state organs, enterprises, and institutions  b. Professional jobs, such as a teacher, doctor, engineer, and writer  c. Nonprofessional jobs  d. Commercial and service industry jobs  e. Jobs related to agriculture, forestry, animal husbandry, fisheries, and water resources production  f. Jobs related to equipment operation or transportation  g. Military  h. Others |
| **8.** **Residency** | a. Rural  b. Urban c. Suburb |
| **9. Household’s monthly capita income (yuan)** | a. < 2000  b. 2000 – 5000  c. 5000 – 10000  d. 10000 – 20000  e. > 20000 |
| **10.** **Smoking** | a. No  b. Quit smoking  c. Yes |
| **11. Alcohol drinking** | a. No  b. Quit drinking  c. No |
| **12. Have you been diagnosed with pelvic floor dysfunction?** | a. Yes, but not yet treated  b. Yes, and has been treated  c. No  d. Unclear |

| **Table S2** **Knowledge questionnaire** | | | |  |
| --- | --- | --- | --- | --- |
| **1.** **Pelvic floor dysfunctions in women include urinary incontinence, bladder emptying disorders, fecal incontinence, bowel emptying disorders, pelvic organ prolapse, sexual dysfunction, and chronic pelvic pain.** | a. True | b. False | c. Unclear | |
| **2. Pelvic floor dysfunction can cause coughing, sneezing, and urine leakage when laughing.** | a. True | b. False | c. Unclear | |
| **3.** **The risk of pelvic floor dysfunction increases with age.** | a. True | b. False | c. Unclear | |
| **4. Pregnancy and childbirth may lead to pelvic floor dysfunction.** | a. True | b. False | c. Unclear | |
| **5. The risk factors for pelvic floor dysfunction include sedentary lifestyles, obesity, long–term restraint of the waist, constipation, and diabetes.** | a. True | b. False | c. Unclear | |
| **6. Due to the strong pressure on the uterus and fetus during pregnancy, including the amniotic fluid, chronic damage to the pelvic floor is inevitable, regardless of whether the delivery is natural or cesarean.** | a. True | b. False | c. Unclear | |
| **7. Pelvic floor function should be tested 42 days after delivery.** | a. True | b. False | c. Unclear | |
| **8.** **Pelvic floor exercises before pregnancy may help prevent postpartum urine leakage.** | a. True | b. False | c. Unclear | |
| **9. Pelvic floor dysfunction can cause physical and mental harm to women.** | a. True | b. False | c. Unclear | |
| **10. Pelvic floor exercises can reduce urine leakage.** | a. True | b. False | c. Unclear | |
| **11. Kegel exercise is one of the most common pelvic floor exercises.** | a. True | b. False | c. Unclear | |
| **12. There are a variety of rehabilitation treatments for pelvic floor dysfunction, including pelvic floor muscle training, low-frequency electrical stimulation therapy, biofeedback therapy, home-use rehabilitation devices (vaginal dumbbells), uterine supports, and surgery.** | a. True | b. False | c. Unclear | |
| **13. Pelvic floor ultrasound is non-invasive, reproducible, radiation-free, and inexpensive.** | a. True | b. False | c. Unclear | |
| **14. Transvaginal ultrasound is the most effective type of pelvic floor ultrasound, followed by transabdominal scan and transperineal scan.** | a. True | b. False | c. Unclear | |
| **15. Early detection and diagnosis of pelvic floor dysfunction can be achieved through pelvic floor ultrasound.** | a. True | b. False | c. Unclear | |
| **16. A pelvic floor ultrasound is essential before pelvic floor surgery.** | a. True | b. False | c. Unclear | |
| **17. Pelvic floor ultrasound can assess the results of pelvic floor surgery, the patient's recovery, and whether pelvic organ prolapse has recurred.** | a. True | b. False | c. Unclear | |

| **Table S3 Attitude questionnaire** | | | | | |
| --- | --- | --- | --- | --- | --- |
| **1. I would like to learn more about pelvic floor dysfunction.** | Strongly Agree | Agree | Neutral | Disagree | Strongly Disagree |
| **2.** **I would like to know how to exercise my pelvic floor muscles.** | Strongly Agree | Agree | Neutral | Disagree | Strongly Disagree |
| **3. Pelvic floor dysfunction can affect my life, mood, and interpersonal relationships.** | Strongly Agree | Agree | Neutral | Disagree | Strongly Disagree |
| **4. Pelvic floor dysfunction can affect my job.** | Strongly Agree | Agree | Neutral | Disagree | Strongly Disagree |
| **5. A woman's pelvic floor dysfunction can negatively affect her relationship with her partner.** | Strongly Agree | Agree | Neutral | Disagree | Strongly Disagree |
| **6. I am willing to undergo a pelvic floor ultrasound if I have pelvic floor dysfunction.** | Strongly Agree | Agree | Neutral | Disagree | Strongly Disagree |
| **7. Which operator is acceptable for performing pelvic floor ultrasound (transperineal scan)?** | a. Female doctors only  b. Both male and female doctors  c. Both male and female doctors, but if the operator is a male doctor, the presence of female medical staff is preferred  d. Both of them are unaccepted | | | | |
| **8. I'm willing to undergo a professional pelvic floor function assessment even if I'm not experiencing symptoms.** | Strongly Agree | Agree | Neutral | Disagree | Strongly Disagree |
| **9. To prevent pelvic floor dysfunction, women of all ages should train their pelvic floor muscles.** | Strongly Agree | Agree | Neutral | Disagree | Strongly Disagree |

| **Table S4 Practice questionnaire** | | | | | | |  |
| --- | --- | --- | --- | --- | --- | --- | --- |
| **1. Have you ever had an assessment of pelvic floor muscle function?** | a. Yes | | | b. No | | |  |
| **2. Have you ever had a pelvic floor ultrasound?** | a. Yes | | | b. No | | |  |
| **3. Have you been proactive in learning about pelvic floor dysfunction?** | a. Yes | | | b. No | | |  |
| **4. If you are concerned about pelvic floor dysfunction, you will be proactive in seeking medical advice.** | a. Yes | | | b. No | | |  |
| **5. How do you exercise your pelvic floor? (Multiple choice)** | a. Kegel exercise  b. Virginal dumbbell  c. Training of pelvic floor muscle repair guided by a medical professional  d. Others  e. Never done it before | | | | | |  |
| **6. How do you usually obtain information about women's healthcare?** | a. Education in the community  b. Internet  c. Newspapers and magazines  d. Radio and television  e. Communication between friends  f. Introduced by healthcare professionals  g. Others  h. Didn't care about this information | | | | | |  |
| **7. You will go for a pelvic floor ultrasound if a friend/relative recommends it to you.** | a. Strongly Agree | b. Agree | c. Neither agree nor disagree/do not know | | d. Disagree | e. Strongly Disagree | |
| **8. Unless you are feeling unwell, I would not undergo a pelvic floor ultrasound.** | a. Strongly Agree | b. Agree | c. Neither agree nor disagree/do not know | | d. Disagree | e. Strongly Disagree | |
| **9.** **You will make an appointment to have related checks if you know someone with pelvic floor dysfunction.** | a. Strongly Agree | b. Agree | c. Neither agree nor disagree/do not know | | d. Disagree | e. Strongly Disagree | |
